# Effect of Microwave Vacuum Freeze-Drying Power on Emulsifying and Structure Properties of Egg White Protein

**DOI:** 10.3390/foods12091792

**Published:** 2023-04-26

**Authors:** Kenan Su, Lili Liu, Xingyu Pan, Shuxing Chen, Xiaodan Zhang, Weiwei Cheng, Baocheng Xu

**Affiliations:** 1 International Joint Laboratory of Food Processing and Quality Safety Control of Henan Province, National Experimental Teaching Demonstration Center for Food Processing and Security, College of Food and Bioengineering, Henan University of Science and Technology, Luoyang 471023, China; sususukenan@163.com (K.S.); m13213598612@163.com (W.C.); luyutting@163.com (B.X.); 2 Food Laboratory of Zhongyuan, Luohe 462300, China

**Keywords:** egg white protein, microwave power, emulsification properties, thermal properties, freeze-drying, structure properties

## Abstract

The study investigated the effects of different microwave vacuum freeze-drying powers (100–500 W) on the emulsifying properties and structural characteristics of egg white protein, which is of great significance in enhancing the added value of EWP and promoting its application. Emulsification analysis revealed that the emulsification performance was significantly influenced by microwave power and reached its maximum at 300 W. Fourier-transform infrared spectroscopy (FT-IR) and sodium dodecyl sulfate–polyacrylamide gel electrophoresis (SDS-PAGE) analyses showed that microwave vacuum freeze-drying treatment altered the secondary structure of EWP without changing its peptide structure. Fluorescence measurements indicated that the maximum fluorescence emission intensity decreased, and the maximum emission wavelength shifted towards blue as the power increased. Particle size, zeta potential, scanning electron microscopy (SEM), and differential scanning calorimetry (DSC) analyses showed that the average particle size of EWP reached the minimum value of 1203.66 nm, the absolute value of zeta potential reached the maximum value of 41.35 mV, and the thermal stability was strongest, with a more uniform and loose structure observed at 300 W. Texture profile analysis (TPA) showed that appropriate power treatment significantly enhanced the chewiness and viscoelasticity of egg white protein. Therefore, appropriate power treatment could effectively improve the emulsifying properties and stability.

## 1. Introduction

China is the world’s largest producer of eggs, accounting for nearly 40% of global production. The global egg market is dominated by whole eggs, which occupy the majority of the market share, while egg white and yolk products have a smaller market share. Egg white products are mainly used in sports nutrition and medical applications. Eggs contain many proteins, phosphorus, lecithin, and vitamins that are necessary for human metabolism. All types of proteins in eggs can be found in the egg white protein (EWP), which is composed of 90% water and 10% protein [1]. Due to eggs having a short shelf life, they are prone to spoilage, which can result in protein waste. Egg white powder has been extensively studied because of its good processing properties and ease of storage. In the production and processing of egg white powder, the drying method is one of the most important factors affecting its functional properties. Hence, the utilization of appropriate drying methods to dry egg whites and develop higher-quality egg white powder is of great market value to expand its applications.

At present, drying EWP has numerous applications in food preparation, such as that of baked, confectionery, and meat products, because of its microbiological safety and lower volume when compared with unshelled or liquid EWP. Egg whites are a food ingredient with multiple functional properties, such as foaming, emulsification, heat-set gelatin, and binding adhesion [2,3]. Yang Yinyue et al. [4] investigated the emulsification behavior of EWP complexes with rhamnolipids, Sun Jun et al. [5] studied the effect of tea polyphenols on the emulsification and antioxidant properties of EWP, Yu Yali et al. [6] explored the changes in emulsification properties by subjecting EWP to extreme acid and alkaline treatments, and Chenying Wang et al. [7] found that glycosylation of modified EWP resulted in enhanced emulsification properties.

Many scholars have used different drying methods to dry egg whites. Our team [8,9] has studied this topic using four drying methods: microwave vacuum freeze-drying, vacuum freeze-drying, hot air drying, and spray drying at the same level, and concluded that microwave vacuum freeze-drying is the most effective. Compared to traditional hot-air drying, microwave drying is faster, more uniform, and more energy-efficient. In recent years, microwave vacuum freeze drying has been investigated as a potential method for obtaining high-quality dried food products [10]. Sun et al. [11] found that microwave treatment significantly increased the protein digestibility of pigeon pea flour by changing starch and protein structure, reducing particle size, and increasing zeta potential, making it a promising approach for improving pigeon pea protein quality and utilization. Liu et al. [12] microwave freeze-drying was better than spray drying in terms of color, emulsion stability, foam stability, water/oil absorption capacity, and thermal stability. Dong et al. [13] suggested microwave vacuum freeze drying of sauerkraut and found that different powers affected the fresh concentration values (flavor intensity) of sauerkraut and that higher powers and vacuum levels had a positive effect on its freshness. Ren et al. [14] studied the effect of glass transition temperature during microwave freeze-drying of mushrooms and found that microwave power had a significant effect on product quality and drying efficiency. Fan et al. [15] optimized microwave vacuum freeze-drying parameters using the response surface method. When the microwave power increases, the drying efficiency also increases significantly. The studies indicate that microwave vacuum freeze-drying significantly reduces drying time and energy consumption, making it a highly efficient method for drying liquid materials. Sai et al. [16] found that microwave treatment could improve the digestibility of soymilk and alter its secondary structure. It demonstrated potential applications in reducing trypsin inhibitor activity and improving the digestibility of soymilk by changing the secondary structures of related proteins. Cao et al. [17] found that microwave heating (MWH), compared to traditional conductive heating, can inhibit the activity of tissue proteinase L and the hydrolysis of the myosin-heavy chain during the degradation stage of fish mince gel, which is beneficial for gel preservation. In summary, microwave vacuum freeze-drying has the potential to dry high-quality EWP powder, but the effect of microwave heating on the natural structure and functional properties of the EWP is unclear.

Current research on the emulsification of EWP focuses on the preparation of emulsions, the investigation of the effect of modifications on EWP, and using protein emulsification as a fat substitute for meat products [18]. The effect of different powers on the emulsifying properties and structural characteristics of egg white protein (EWP) was investigated using microwave vacuum freeze-drying technology. This study is of great significance for the production of high-quality egg white powder. High-quality EWP powder has stronger emulsifying properties and a more stable system, with broad application prospects in fields such as food, medicine, cosmetics, and others.

## 2. Materials and Methods

### 2.1. Material

The fresh eggs were purchased from Dazhang supermarket in Luoyang, China. All chemicals and pure standards were purchased from Macklin (Shanghai, China).

### 2.2. Preparation of Microwave Vacuum Freeze-Dried (100~500 W) EWP Powder

A multifunctional microwave dryer was developed by Duan et al. [19]. Fresh eggs were cleaned of surface stains and separated into egg whites and yolks. The egg white was clarified by filtration through filter paper, removing suspended impurities and insoluble substances, resulting in an egg white solution. The egg white solution was stirred and naturally fermented at room temperature 25 °C for 48 h for desugarization. Next, the egg white solution was pasteurized at 45 °C for 30 min under 100 KPa before drying. The egg white protein solution was placed on a plate and pre-chilled (−18 °C) for 3 h in the refrigerator freezer. Before starting the vacuum pump, the blower and cooler were cleaned, and the cold trap temperature was reduced to −40 °C. The freeze-drying machine parameters were set at a fixed vacuum of 160 Pa, loading capacity of 200 g, and different microwave powers (100 W, 200 W, 300 W, 400 W, 500 W) for design experiments. After 6 h of microwave vacuum freeze-drying, the material trays were removed and weighed every half hour, and drying was stopped when the difference between two consecutive measurements did not exceed 0.01 g. The protein concentration of EWP after microwave vacuum freeze-drying was determined to be 0.264 g/100 mL by the Kjeldahl method.

### 2.3. Preparation of EWP Emulsions

The method was slightly modified by referring to Wang [20]. A total of 0.2 g of EWP powder treated with different powers was weighed and dissolved in 100 mL of water. Stirring was carried out using a high-speed mixer (JJ-1, China Precision Instrument Factory) at 1000 rpm for 1 h at 25 °C. A total of 15 mL of the stirred protein solution was then mixed with 5 mL of soybean oil (purchased from Dazhang Supermarket, Luoyang, China) and emulsified at 13,500 r/min for 2 min under a high-speed emulsifying homogenizer (FSH-2A, Jintan City Kexing Instrument Factory, Jintan, China) to obtain the emulsion. Emulsions were mainly used for zeta potential determination and particle size determination.

### 2.4. Determination of Emulsification Activity Index (EAI) and Emulsion Stability Index (ESI)

EAI and ESI of the EWP powder were measured based on the method of Xue Xiuheng [21] with some modifications. The protein powder sample (0.2 g) was dissolved in distilled water (100 mL). The suspension (30 mL) was then added to soybean oil (10 mL) and homogenized (FSH-2A, Jintan City Kexing Instrument Factory, China) for 3 min at room temperature. The prepared emulsion was mixed and homogenized with 5 mL of SDS at a concentration of 0.1%. A UV-Vis spectrophotometer (722 N, Shanghai, China) was used to record the absorbance of the solution instantly (*A*_0_) and at 10 min (*A*_10_) after the emulsion against SDS solution (0.1% *w*/*v*). The constant 2.303 is a constant with a natural logarithmic base, and the constant 0.25 indicates that a 4-fold dilution is required during the *EAI* determination. The control group with 0 W microwave power under the same conditions was prepared using samples that had been vacuum freeze-dried. *EAI* and *ESI* were calculated as follows:

(1) $$EAI\left(m^{2}/g\right)=\frac{A_{0}\times 2\times 2.303}{0.25}$$

(2) $$ESI\left(\min\right)=\frac{A_{0}}{A_{10}-A_{0}}\times 10$$

### 2.5. Zeta Potential Determination

The emulsion prepared in Section 2.3 was taken as a sample. The samples were diluted 50 times with 10 mmol/L pH 7.0 phosphate buffer, the relative refractive index of the emulsion was set to 1.450, and it was measured three times at room temperature using a zeta potential analyzer (Malvern Instruments Co., Ltd., London, UK). The control group with 0 W microwave power under the same conditions was prepared using samples that had been vacuum freeze-dried.

### 2.6. Particle Size Determination

The emulsion prepared in 2.3 was taken as a sample. The particle size of the samples was determined using a laser particle size meter (Malvern Instruments Co., Ltd., UK), with measurements taken in units of nanometers (nm). Emulsions of 100 μL were diluted 50 times with distilled water and equilibrated at 25 °C for 2 min. When the test was started, each sample was measured three times in parallel. The control group with 0 W microwave power under the same conditions was prepared using samples that had been vacuum freeze-dried.

### 2.7. Fourier Infrared Spectroscopy (FT-IR)

FT-IR spectroscopy was carried out to analyze the chemical structure and secondary structure content of the protein samples. First, the dried protein sample was mixed and pressed with KBr powder in a ratio of 1:200. Then it was analyzed by FT-IR spectrophotometer (Equinox55, Bruker Co., Berlin, Germany) at 500–4000 cm^−1^ wavelength using scan 16 with a resolution of 4 cm^−1^. In addition, the infrared spectra of the amide I band (1600–1700 cm^−1^) were analyzed using Peakfit software to obtain the secondary structure contents of the EWP samples based on the sub-peak area. The control group with 0 W microwave power under the same conditions was prepared using samples that had been vacuum freeze-dried. When the test was started, each sample was measured three times in parallel.

### 2.8. Sodium Dodecyl Sulfate–Polyacrylamide Gel Electrophoresis (SDS-PAGE)

The sodium dodecyl sulfate–polyacrylamide gel electrophoresis (SDS-PAGE) analysis of EWP was performed following the discontinuous buffer system of Laemmli [22] at 5% stacking gel and 10% separating gel by using an electrophoresis equipment (DYC-Mini4, Yucheng, China). The molecular weight markers (Fermentas, St. Leon-Rot, Germany) were run simultaneously to determine the molecular weight. The gels were stained with a solution containing 1% Coomassie Brilliant Blue R-250, 30% methanol, and 8% acetic acid (*v*/*v*). The control group with 0 W microwave power under the same conditions was prepared using samples that had been vacuum freeze-dried.

### 2.9. Scanning Electron Microscopy (SEM) Analysis

Retrieve the sample and perform gold sputtering on it. Then, use conductive silver adhesive to affix the sample onto the scanning electron microscope sample stage. Then, the sample was placed under a scanning electron microscope (Shimadzu, Japan), set with an accelerating voltage of 20 kV and a vacuum pressure of 400 Pa, and the microstructure of the sample was observed under a magnification of ×2000 and ×5000. The control group with 0 W microwave power under the same conditions was prepared using samples that had been vacuum freeze-dried.

### 2.10. Endogenous Fluorescence Spectroscopy Analysis

According to the method of Zhao et al. [23], 120 μL of the emulsion sample was dissolved in 8 mL of 0.01 mol/L pH 7.0 phosphate buffer solution shaken thoroughly, and the excitation wavelength was set at 290 nm, and a high voltage of 800 V was used to scan the evanescence spectrum in the range of 300–400 nm with the excitation and emission slit widths of 5 nm. The control group with 0 W microwave power under the same conditions was prepared using samples that had been vacuum freeze-dried. When the test was started, each sample was measured three times in parallel.

### 2.11. Differential Scanning Calorimetry (DSC) Analysis

Determination was performed according to Liu et al. [24]. The thermal properties of EWP treated with different microwave powers were analyzed by DSC. The temperature range was 30–110 °C, the heating rate was 10 °C/min, the sample dosage was 5–8 mg each time, and the nitrogen flow rate was 60 mL/min. The control group with 0 W microwave power under the same conditions was prepared using samples that had been vacuum freeze-dried. When the test was started, each sample was measured three times in parallel.

### 2.12. Texture Profile Analysis (TPA) Analysis

Emulsion gels were prepared and subjected to texture profile analysis (TPA) according to the method of Han [25]. The measurements were conducted in TPA mode using a P50 probe with a pre-test speed of 1.0 mm/s, a test speed of 1.0 mm/s, and a post-test speed of 1.0 mm/s. The interval between the two measurements of the probe was 1.00 s, and the compressive deformation was set to 50% of the sample. The thixotropic force was 1.0× *g*, and the detection temperature was room temperature. The texture properties, including hardness, adhesiveness, chewiness, resilience, and water loss rate, were recorded. Each sample was measured three times in parallel. The control group with 0 W microwave power under the same conditions was prepared using samples that had been vacuum freeze-dried.

### 2.13. Statistical Analysis

The measurements involved in the experiment were made in three replicates, and the experimental data were plotted using Origin 8.5 software. Statistical significance was analyzed using DPS 7.5 software. Data analysis was conducted using Omnic 2021 and PeakFit 8.2.0 software.

## 3. Results

### 3.1. EWP Emulsification Properties Analysis

As shown in Table 1, as the microwave power increased, the emulsifying activity index (EAI) and emulsion stability index (ESI) of EWP powder significantly increased, reaching their maximum values of 61.35 m^2^·g^−1^ and 33.53 min at 300 W (*p* < 0.05). However, as the microwave power continued to increase, both EAI and ESI began to decrease significantly (*p* < 0.05). This was due to the moderate microwave effect disrupting hydrogen bonds and van der Waals forces in EWP, causing its structure to become looser and exposing the internal functional groups, which improved protein adsorption to fat. Yalcin et al. [26] found that the emulsification activity of microwave-treated wheat gluten proteins was also improved, which was consistent with the results of this study. Additionally, as the microwave power increased, the average particle size significantly decreased, and the absolute value of the zeta potential significantly increased. When the power was 300 W, the average particle size reached its minimum value of 1203.66 nm, and the absolute value of the zeta potential reached its maximum value of 41.35 mV. As the power continued to increase to 500 W, the average particle size began to significantly increase, and the absolute value of the zeta potential began to significantly decrease. This was likely due to the fact that too high a microwave power caused the proteins to aggregate and form larger protein molecules, leading to decreased emulsion stability [27,28].

**Figure 1:**
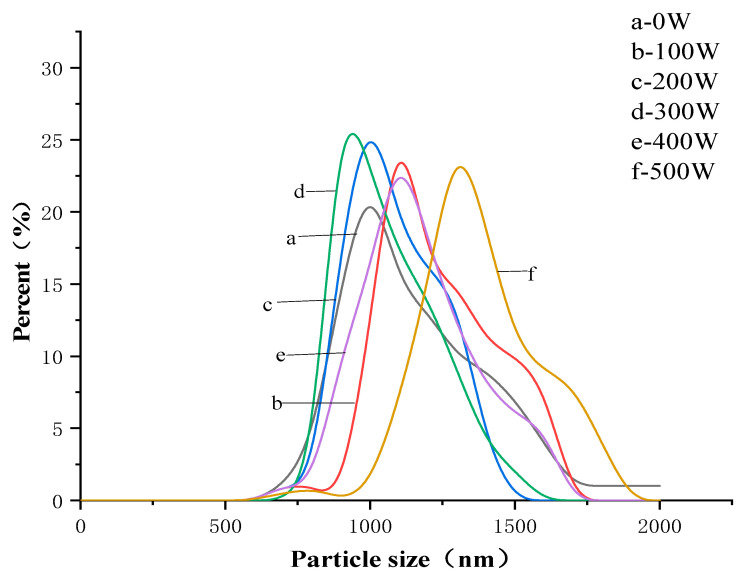
Particle size distribution of EWP treated at different powers.

From Figure 1, it can be observed that the particle size distribution of EWP powder varies under different power treatments. By examining the peaks in Figure 1 in conjunction with Table 1, it can be analyzed that the peak at 300 W is closest to 0 nm, indicating the smallest average particle size. Moreover, when the power is set to 300 W, the width of the particle size distribution peak is the smallest. A smaller width of the peak in the particle size distribution indicates greater uniformity in the size of the protein, implying stronger stability of the protein. This result is consistent with the findings from DSC and SEM in this study.

### 3.2. Structural Properties of EWP

#### 3.2.1. FT-IR Spectroscopic Analysis

**Figure 2:**
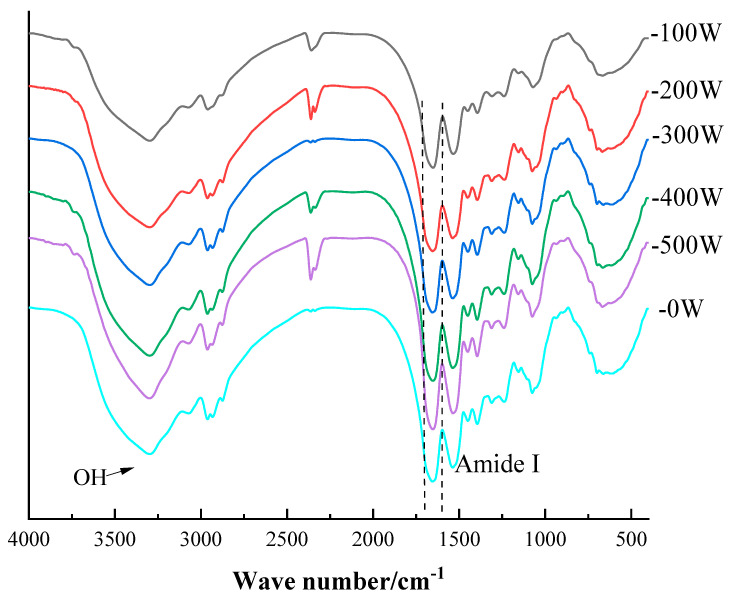
The FT-IR curves for different power.

The study of the secondary structure information of EWP treated with different microwave powers and vacuum drying was performed using FT-IR. Changes in the peak position of amides I (1700–1600 cm^−1^), II (1530–1550 cm^−1^), and III (1260–1300 cm^−1^) indicate the transformation of protein structures [29]. The structure of the protein molecule determines its function. Analysis of peak position changes in Figure 2 showed that the amide I band of EWP powder treated with 300 W was red-shifted in the direction of a high wave number compared to the other power levels of the EWP amide I band. The intensity of the OH absorption peak gradually increased with increasing power, and when the power reached 500 W, the intensity of the absorption peak decreased. This was likely due to excessive power causing OH to fracture. The results indicated that the microwave drying power caused a shift change in the peak position of the secondary structural unit composition in the protein molecule.

As shown in Table 2, with the increase in microwave power, the content of each composition in the secondary structure changed significantly. The content of the secondary structure can be analyzed by combining the peak width and peak intensity of the amide band in the Fourier-transform infrared spectrum. With the increase of power, the relative content of α-helix significantly increased and reached the maximum value of 29.90% at 200 W, followed by a gradual decrease. When the power increased from 400 W to 500 W, the relative content of α-helix significantly decreased by 12.54% (*p* < 0.05). As the power increased from 100 W to 200 W, the relative content of the β-sheet decreased. From 200 W to 400 W, the content increased, but it decreased significantly from 400 W to 500 W. The relative content of β-fold reached the maximum value of 42.27% at 400 W (*p* < 0.05). The hydrogen bond between the carbonyl and amino groups of the polypeptide chain is the main force to maintain the stability of the α-helix [30]. The reason for this phenomenon may be that the temperature in the drying chamber increased with the increase in power during microwave drying, and the molecular structure unfolded, destabilizing the alpha-helix structure and leading to a decrease in the ratio. When the microwave power was 500 W, the α-helix content secondary structure of the egg protein powder dropped to the lowest. The reason for this may be due to the high microwave power and radiation energy, resulting in a non-spiral phenomenon in the EWP powder. The effect of microwave power 300 W on EWP powder β-fold content increased significantly. The reason may be that the proper microwave power radiation makes the protein structure more diffused, resulting in a β-rotation angle of transformation and the structure increased [31]. The unfolding of the protein structure facilitates the exposure of the groups wrapped inside the protein molecule and the enhancement of the surface activity, thus improving its emulsification properties. This was in line with the previous section where EAI and ESI were maximum when the power was 300 W. 

#### 3.2.2. SDS-PAGE Analysis

**Figure 3:**
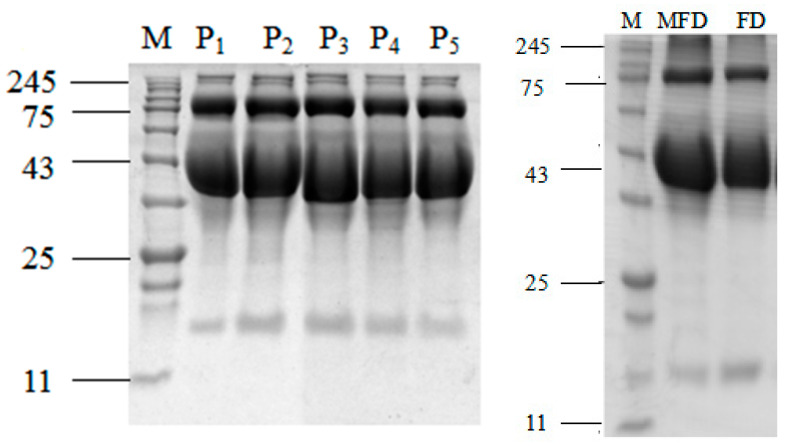
SDS-PAGE of egg white powder protein with different microwave power. M is the standard protein. P_0_ to P_5_ are EWP powders treated at 0 W, 100 W, 200 W, 300 W, 400 W, and 500 W, respectively. MFD is P_1_, and FD is P_0_.

The main proteins in egg white protein are ovalbumin (45 kDa), ovotransferrin (72–90 kDa), and lysozyme (11–17 kDa). As can be seen from Figure 3, most of the bands in each lane of the A, B, and C plots appeared at 75 kDa, 43 kDa, and 16 kDa. This indicated that no significant changes in the primary structure of EWP in terms of protein number were found after the EWP was freeze-dried by microwave vacuum. The reason for this may be that the microwave vacuum freeze-drying treatment could not destroy the peptide structure of EWP. The color of the MFD band around 245 kDa was darker than that of the FD band, indicating that EWP tended to aggregate after microwave treatment, and a certain degree of aggregation was beneficial for gel properties. The lysozyme content in EWP was lower at 100 W and 500 W compared to other powers. The reason for this was a long heating time at low power and the high heat temperature of the egg white protein at high power, resulting in the denaturation and aggregation of EWP lysozyme. This was also responsible for the reduced emulsification of egg white powder.

#### 3.2.3. SEM Analysis

**Figure 4:**
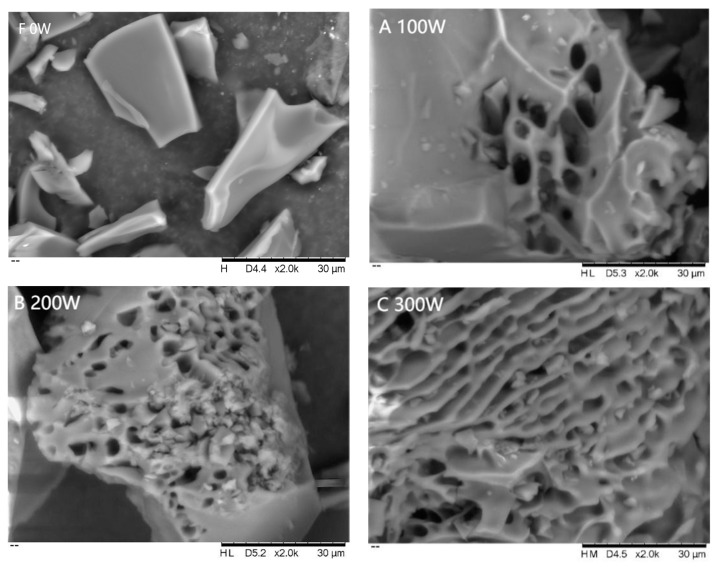

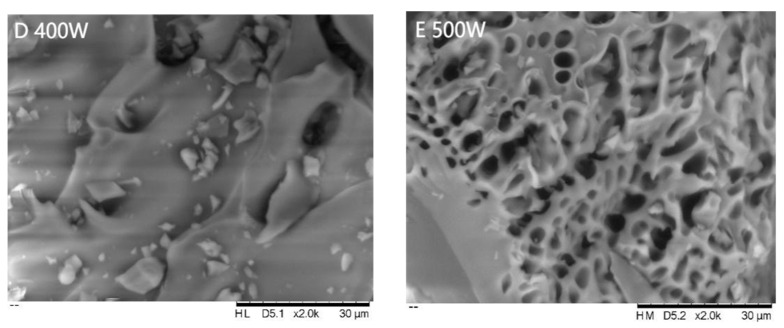
SEM of EWP powder after different microwave treatment (×2000).

**Figure 5:**
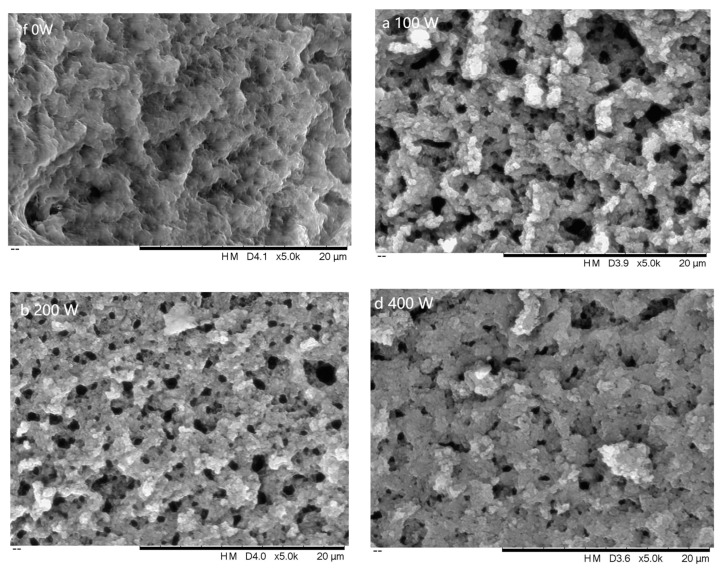

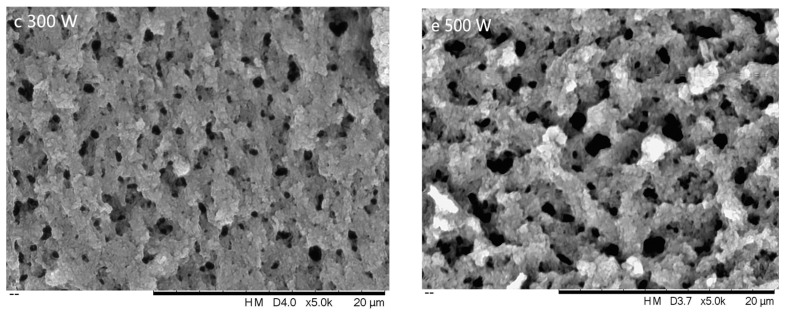
SEM of EWP powder after different microwave treatment (×5000).

By comparing the micrographs shown in Figure 4A and Figure 5a, we found that the EWP powder structure was relatively regular and dense with fine local pores and a surface full of particles of uneven size (×2000), and the pore and particle distribution was uneven (×5000) under 100 W treatment. As the power increased, the pores became denser, the structure became more uniform, and the surface particles decreased. However, at higher powers (400 W and 500 W), the structure became uneven, with larger pores and more surface particles. This may be due to enhanced protein interactions resulting from the frictional effect of polar molecules at overpowered microwave treatment. However, by examining all ten figures, we observed that at 300 W, the pore structure was more uniform and regular. This is consistent with the finding that the average particle size was the smallest and the absolute value of the zeta potential was the largest at 300 W, indicating a more stable system. The molecular flexibility of protein molecules [32] may be responsible for this phenomenon, as it makes them more likely to unfold and change shape under appropriate microwave irradiation. Overall, suitable microwave irradiation could enhance the cross-linking of protein powder, which contributes to a more stable structure.

#### 3.2.4. Endogenous Fluorescence Spectroscopy Analysis

**Figure 6:**
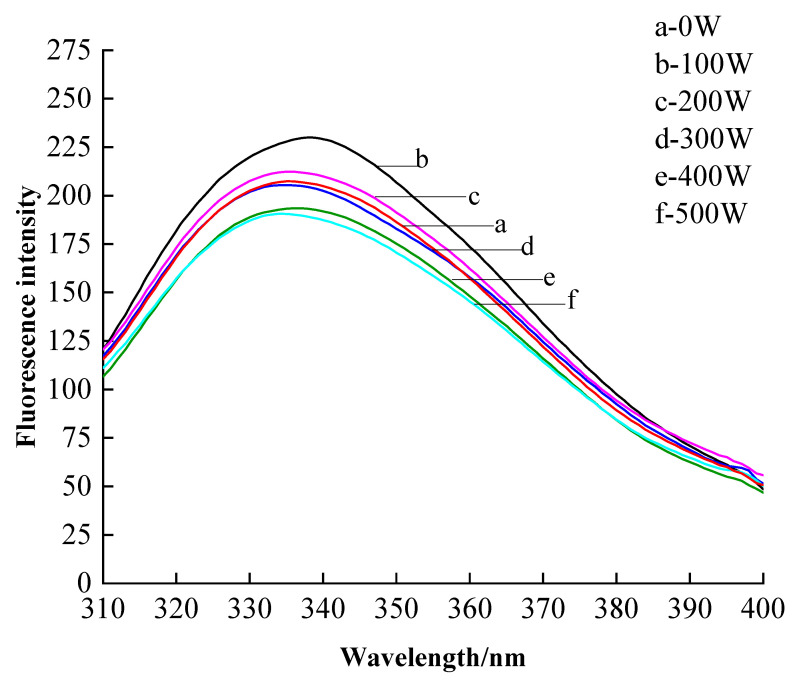
The FS curves for different power. a. 0 W, b. 100 W, c. 200 W, d. 300 W, e. 400 W, and f. 500 W.

Endogenous fluorescence spectra can determine protein conformational changes and reflect changes in side chains by analyzing spectral changes. The endogenous fluorescence of proteins mainly originates from tryptophan which is more sensitive to microenvironmental changes. The surface-exposed tryptophan residues of proteins can act as surfactants during the emulsification process, reducing interfacial tension and stabilizing emulsions, which is closely related to the emulsifying properties of proteins [33]. Figure 6 shows the effect of different microwave treatments on the endogenous fluorescence of EWP powder. As the microwave power increased, the fluorescence intensity gradually decreased. The maximum fluorescence intensity at 500 W was 190.54 compared to 229.93 at 100 W, a decrease of 13.92%. λ_max_ shifted blue from 343.07 nm to 330 nm. This might be due to the polymerization precipitation of protein molecules by high microwave power so that the Trp residues exposed on the surface of protein molecules were wrapped inside the protein [34]. When the power was 500 W, the fluorescence intensity decreased. Combined with the results of scanning electron microscopy and the increase in average particle size, it was found that EWP had undergone aggregation.

#### 3.2.5. DSC Analysis

DSC is commonly used in the thermodynamic study of the thermal denaturation process of proteins. The thermal denaturation process occurs when a protein absorbs energy by heat, hydrogen bonds break, and the protein molecule unfolds into a disordered structure. The peak temperature (Tp) represents the degree of thermal stability and aggregation of the protein. The size of the enthalpy (ΔH) usually reflects the change in protein structure; the lower the enthalpy, the more complete the unfolding of the protein structure.

**Figure 7:**
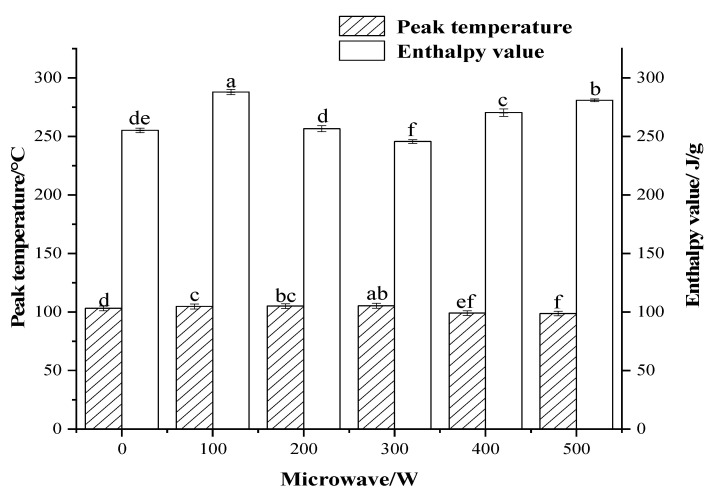
Effect of microwave power on thermodynamic properties of EWP. Different letters indicate a significant difference at a 95% confidence level.

As shown in Figure 7, the peak temperature of egg white protein increased and then decreased with increasing power, with insignificant changes between 100 W and 300 W. The decrease in peak temperature between 400 W and 500 W indicated that the protein molecules are more sensitive to heat. The enthalpy value of egg white protein significantly decreased with the increase of power, reaching the minimum value of 245.70 J/g at 300 W, and then increased with the further increase of power. The decrease in enthalpy indicated that the degree of protein denaturation increased with increasing power, the side chains of the protein structure unfolded, and the gel hardness increased. It indicated that the protein molecules unfolded most completely at this time, the α-helix structure was destroyed, the sulfhydryl groups inside the molecules were exposed, and the surface hydrophobicity increased [35]. The increase in enthalpy between 400 W and 500 W may be due to the high drying efficiency and short drying time, where protein molecules do not unfold significantly and more energy is required for structural stabilization. It may be due to the high temperature at the end of drying, where non-covalent bonding between protein molecules aggregates, resulting in higher energy requirements. Zehra et al. [36] found that microwave treatment had a significant impact on the physicochemical properties of carob flour. They found that the total phenolic content, total antioxidant activity, browning index, and UV absorption of carob flour increased.

#### 3.2.6. TPA Analysis

As can be seen from Table 3, the emulsion gel hardness of EWP increased and then decreased as the microwave power increased. When the microwave power was increased to 300 W, the emulsion gel hardness of EWP reached a maximum value of 369.62 ± 4.19 g and was significantly different compared to other powers (*p* < 0.05). The emulsion gel hardness of EWP was 353.55 ± 3.92 g and 289.46 ± 5.34 g at 400 and 500 W, respectively, which was significantly lower than that at 300 W. As the power increased, the bonding force and chewiness both tended to increase and then decrease. The change in bonding force was not significant, while the change in chewiness was significant (*p* < 0.05). The resilience decreased and then increased with increasing power and did not change significantly between 200 W and 300 W. The interaction between non-covalent bonds is the key to maintaining the spatial structure of protein molecules. An increase in microwave power increases the temperature at the end of drying; the greater the degree of molecular unfolding, the easier the inter-molecular aggregation reactions, facilitating the formation of a regular protein network structure, thus contributing to the increase in the hardness of the EWP emulsion gel. Appropriate microwave power treatment of EWP emulsion gels can enhance their gel hardness, viscoelasticity, and chewiness and reduce their water loss. Jiang et al. [37] found that microwave heating promotes glycosylation of α-lactalbumin, resulting in higher levels of browning, free amino groups, and reaction rates. The glycosylation products showed increased surface hydrophobicity, antioxidant capacity, and α-glucosidase inhibitory activity. This facilitates subsequent product development regarding EWP.

#### 3.2.7. Correlation Analysis

**Figure 8:**
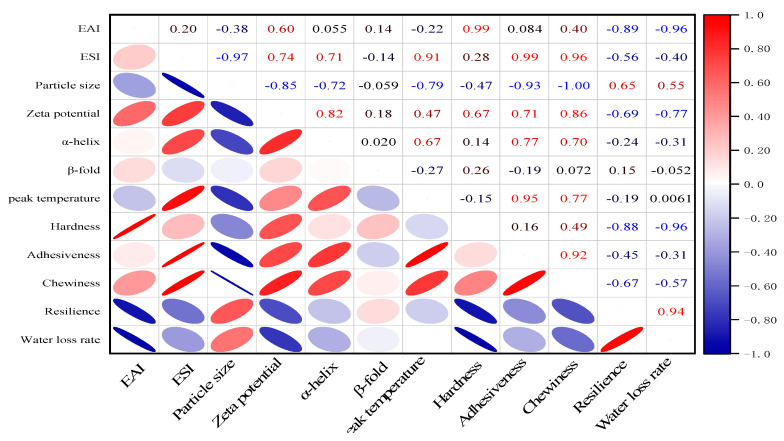
Correlation between emulsifying properties and structural characteristics changes of EWP. The ellipse represents the degree of correlation.

According to Figure 8, EAI was positively correlated with hardness and negatively correlated with viscoelasticity and water loss, with correlation coefficients of 0.99, −0.89, and −0.96, respectively. ESI was negatively correlated with the average particle size but positively correlated with the zeta potential, α-helix folding, and thermal denaturation temperature, with correlation coefficients of −0.97, 0.74, 0.71, and 0.91, respectively. As the ESI increased, the zeta potential decreased, and the greater the absolute value of the zeta potential, the higher the stability and thermal stability of the system. The emulsifying properties and internal structural changes of EWP were closely related.

## 4. Conclusions

Through SDS-PAGE, we found that microwave vacuum freeze-drying did not break down the peptide structure of EWP, but it had an effect on the secondary structure and could make the porous structure of EWP more uniform and regular. When the microwave power was 300 W, the average particle size of the protein powder emulsion was the smallest, and the absolute value of the zeta potential was the largest. The emulsion gel made from EWP was the hardest and had the best chewiness and viscoelasticity. The microstructure of the powder was looser, the thickness of the emulsion droplets was more uniform, and the thermal stability was greater at 300 W. Microwave vacuum freeze-drying had a significant impact on the emulsification properties of EWP powder. Increasing the microwave power changed the molecular conformation of the protein, which affected the emulsification activity and emulsion stability of the powder. However, when the microwave power was too high, the interaction between the proteins was enhanced, and the proteins were less stretched, making the protein structure rougher and less homogeneous. The droplet diameter of the emulsion was larger and less stable. Therefore, treatment with 300 W microwave power can improve the emulsification properties and system stability of EWP powder. The correlation analysis showed that there was a significant correlation between the emulsification properties and structural characteristics of EWP. This study can provide a reference for the production of EWP powder using microwave vacuum freeze-drying.

## Figures and Tables

**Table 1:** Emulsification properties of egg albumin at different power.

Microwave Power/W	EAI/m^2^·g^−1^	ESI/min	Particle Size/nm	Absolute Value of the Zeta Potential/mV
0	35.27 ± 0.29 ^e^	26.51 ± 0.84 ^c^	1507.23 ± 14.88 ^d^	35.57 ± 0.89 ^d^
100	33.40 ± 0.31 ^f^	26.92 ± 1.14 ^d^	1619.01 ± 16.52 ^c^	34.53 ± 1.33 ^e^
200	51.19 ± 0.59 ^c^	29.91 ± 1.13 ^b^	1494.11 ± 38.65 ^e^	37.93 ± 0.99 ^c^
300	61.35 ± 0.58 ^a^	33.53 ± 0.62 ^a^	1203.66 ± 13.66 ^f^	41.35 ± 1.61 ^a^
400	58.88 ± 0.86 ^b^	23.17 ± 0.95 ^e^	1668.33 ± 28.82 ^b^	39.37 ± 1.38 ^b^
500	48.17 ± 0.25 ^d^	20.09 ± 0.88 ^f^	1923.67 ± 16.49 ^a^	29.57 ± 0.33 ^f^

All the data are expressed as mean ± SD, and the letters within the same line denote significant differences (*p* < 0.05).

**Table 2:** Secondary structure content of EWP powder with different microwave treatments.

Microwave Power/W	Secondary Structure Content/%			
	α-helix	β-fold	β-rotation angle	Irregularconvolution
0	37.19 ± 0.02 ^a^	32.71 ± 0.02 ^f^	15.67 ± 0.04 ^c^	14.27 ± 0.01 ^f^
100	28.28 ± 0.01 ^d^	40.81 ± 0.04 ^c^	11.89 ± 0.04 ^d^	18.10 ± 0.03 ^c^
200	29.90 ± 0.04 ^b^	33.29 ± 0.03 ^e^	19.44 ± 0.02 ^b^	17.34 ± 0.02 ^e^
300	28.79 ± 0.03 ^c^	41.58 ± 0.01 ^b^	25.83 ± 0.03 ^a^	18.21 ± 0.07 ^b^
400	26.75 ± 0.01 ^e^	42.27 ± 0.05 ^a^	11.47 ± 0.04 ^f^	17.51 ± 0.03 ^d^
500	16.21 ± 0.02 ^f^	38.71 ± 0.02 ^d^	11.52 ± 0.05 ^e^	19.25 ± 0.01 ^a^

All the data are expressed as mean ± SD, and the letters within the same line denote significant differences (*p* < 0.05).

**Table 3:** Effect of microwave power on texture characteristics of egg white powder.

Microwave Power/w	Hardness/g	Adhesiveness	Chewiness	Resilience	Water Loss Rate/%
0	307.28 ± 2.47 ^c^	0.689 ± 0.02 ^e^	201.271 ± 2.87 ^b^	0.076 ± 0.002 ^b^	34.21 ± 0.07 ^b^
100	226.57 ± 4.44 ^f^	0.733 ± 0.03 ^bc^	157.334 ± 2.15 ^d^	0.077 ± 0.001 ^a^	36.43 ± 0.08 ^a^
200	299.77 ± 5.01 ^d^	0.751 ± 0.02 ^b^	176.342 ± 5.44 ^c^	0.067 ± 0.003 ^e^	31.07 ± 0.10 ^d^
300	369.62 ± 4.19 ^a^	0.764 ± 0.03 ^a^	220.737 ± 3.31 ^a^	0.064 ± 0.002 ^f^	29.14 ± 0.09 ^f^
400	353.55 ± 3.92 ^b^	0.7 ± 0.02 ^d^	152.583 ± 4.13 ^e^	0.069 ± 0.001 ^d^	29.67 ± 0.11 ^e^
500	289.46 ± 5.34 ^e^	0.675 ± 0.01 ^f^	114.847 ± 2.51 ^f^	0.072 ± 0.002 ^c^	33.92 ± 0.07 ^c^

All the data are expressed as mean ± SD, and the letters within the same line denote significant differences (*p* < 0.05).

## Data Availability

Data is contained within the article.
